# Retinal Fundus Image Enhancement Using the Normalized Convolution and Noise Removing

**DOI:** 10.1155/2016/5075612

**Published:** 2016-09-04

**Authors:** Peishan Dai, Hanwei Sheng, Jianmei Zhang, Ling Li, Jing Wu, Min Fan

**Affiliations:** ^1^Department of Biomedical Engineering, School of Geosciences and Info-Physics, Central South University, Changsha 410083, China; ^2^Department of Education and Law, Hunan Women's University, Changsha 410004, China

## Abstract

Retinal fundus image plays an important role in the diagnosis of retinal related diseases. The detailed information of the retinal fundus image such as small vessels, microaneurysms, and exudates may be in low contrast, and retinal image enhancement usually gives help to analyze diseases related to retinal fundus image. Current image enhancement methods may lead to artificial boundaries, abrupt changes in color levels, and the loss of image detail. In order to avoid these side effects, a new retinal fundus image enhancement method is proposed. First, the original retinal fundus image was processed by the normalized convolution algorithm with a domain transform to obtain an image with the basic information of the background. Then, the image with the basic information of the background was fused with the original retinal fundus image to obtain an enhanced fundus image. Lastly, the fused image was denoised by a two-stage denoising method including the fourth order PDEs and the relaxed median filter. The retinal image databases, including the DRIVE database, the STARE database, and the DIARETDB1 database, were used to evaluate image enhancement effects. The results show that the method can enhance the retinal fundus image prominently. And, different from some other fundus image enhancement methods, the proposed method can directly enhance color images.

## 1. Introduction

Retinal fundus images provide rich information of pathological changes which may indicate diseases such as arteriosclerosis, diabetes, hypertension, stroke, and cardiovascular disease [1]. These images are widely used for diagnosis of related disease. However, the retinal fundus images are usually with low contrast, uneven illumination, and blur of the details due to the complex imaging environments. The purpose of retinal fundus image enhancement is to improve the contrast and highlight the retinal vessels [2].

There are many image enhancement methods. Histogram equalization (HE) [3] is a popular method to improve image contrast, but the decreasing of the gray levels may result in the loss of image details. To overcome this deficiency, contrast limited adaptive histogram equalization (CLAHE) is proposed [4]. But the CLAHE method may produce artificial boundaries at the region which has an abrupt change in the gray levels. Due to the special characteristic, enhancement of retinal fundus image needs specific design. Setiawan et al. [5] demonstrated that CLAHE method is suitable for improving the retinal image quality. Ashiba et al. [6] proposed an image enhancement method based on a wavelet-based homomorphic filter, which can improve image contrast and dynamic range. However, it is difficult to find a suitable structuring element for the morphological filter. Oh and Hwang [7] proposed an image enhancement method based on morphology-based homomorphic filter and differential evolution algorithm. But the target image region should be input firstly, which may limit the scope of its application. U. Qidwai and U. Qidwai [8] proposed a retinal image enhancement method based on the blind deconvolution approach using maximum likelihood estimation. And this deconvolution approach showed a promising result, which may be used for clinical application. Fraz et al. [9] used a 2D Gabor filter to enhance retinal fundus image, which could detect the vessels in multidirection. However, it included some sensitive parameters. Bai et al. [10] proposed an image enhancement method based on multiscale top-hat transformation. In this method, the image details were well enhanced, but the contrast is not improved effectively. Rampal et al. [11] used a complex diffusion-based shock filter for retinal image smoothing and contrast enhancement. And this method outperformed other methods on the DIARETDB1 database. Liao et al. [12] used a hybrid model of multiscale top-hat transformation and histogram fitting stretching to enhance retinal fundus image, which could enhance the contrast of the retinal image effectively and highlight the retinal vessels well. But some parameters have to be set cautiously in the part of histogram fitting stretching.

In this paper, a retinal image enhancement method was proposed to avoid producing artificial boundaries, abrupt changes in color levels, and the loss of image detail. First, we used normalized convolution with a domain transform to obtain an image containing the basic information of the original image. Then, the image containing the basic information was fused with the original image to enhance vessels and detail of the retinal fundus image. Lastly, the fused image went through denoising filters to achieve image enhancement.

The rest of this paper is organized as follows. In Section 2, the proposed method is introduced in detail. In Section 3, experimental results of three well-known databases are used to demonstrate the effectiveness of the proposed method. Finally, in Section 4, the conclusion is presented.

## 2. Materials and Methods

It is very difficult to enhance retinal fundus images directly due to the uneven illumination of imaging characteristic. So different from commonly used image enhancement methods which enhance retinal fundus image directly, the proposed method firstly obtained an image with the basic information of the background through some process of the original image and then fused this image with the original image to suppress background to achieve the goal of image enhancement. As the image enhancement process is realized by background suppression, the proposed method may reduce occurrence probability of artificial boundaries, abrupt changes in color levels, and the loss of image detail.

We used the normalized convolution with a domain transform proposed by Gastal and Oliveira [13] to obtain a background image with the basic information and then fused the image with the original image to achieve retinal fundus image enhancement. Lastly, the fused image was denoised by the fourth order PDEs [14] and the relaxed median filter [15].

### 2.1. Normalized Convolution with Domain Transform

Normalized convolution [16] uses neighbor information to model the image pixel of the original image. In [13], normalized convolution was used to obtain edge-preserving smoothed images with a domain transform box kernel. But if doing some variation for the parameters, the smoothing method can produce an image with the basic information of the background of the retinal fundus image, which does not focus on edge-preserving. The processing procedures are as follows.

Let *I* represent the original retinal fundus image, and *p* = (*x*_*p*_, *y*_*p*_) denotes the position of a pixel in the original image *I*, and *I*(*p*) = (*r*_*p*_, *g*_*p*_, *b*_*p*_) denotes the pixel value in the 2D RGB color image. *D*(*Ω*) denotes the domain of *p*, that is, *p* ∈ *D*(*Ω*). Then the normalized convolution with a domain transform is as follows [13]:

(1) Jp=1Kp∑q∈DΩIqHtp^,tq^,Kp=∑q∈DΩHtp^,tq^,

where *K*_*p*_ is the normalization factor for *p*. And $t(\hat{p})$ is a domain transform.

(2) $$\begin{array}{c} t\left(\;\hat{p}\;\right)=ct\left(\;p\;\right)\overset{p}{\underset{0}{\int }}1+\frac{\delta_{s}}{\delta_{r}}\overset{3}{\underset{k=1}{\sum }}\left|\;I_{k}^{\prime }\left(\;x\;\right)\;\right|dx, \end{array}$$

where *I*_*k*_′(*x*) is the derivative of *k*th channel of the retinal color image and *δ*_*s*_ and *δ*_*r*_ are standard deviation of typically Gaussian spatial and range filters [17].

As the domain transform *ct*(*x*) is monotonically increasing, an efficient moving-average approach [18] is used to perform NC with a box filter $H(t(\hat{p}),t(\hat{q}))$. The box kernel is defined as follows [13]:

(3) Htp^,tq^=δtp−tq≤r,r=δH3,δHi=δH32N−i4N−1,

where *δ*_*H*_*i*__ is the standard deviation for the kernel used in the *i*th iteration, *N* is the total number of iterations, and *δ*_*H*_ is the standard iteration of the desired kernel.

### 2.2. Image Fusion

In the above section, we obtained the background image with basic information. In order to obtain an enhancement result, we fuse the background image with the original image. The fusing result is defined as

(4) $$\begin{array}{c} P=I-a\left(\;J-I\;\right), \end{array}$$

where *P* is the enhancement result and *J* and *I* are the image with the basic information of the background (represented as *J*(*p*) in Section 2.1) and the original image, respectively. And *a* is a parameter factor which could determine the contrast of the image.

### 2.3. Image Denoising

Noise may be amplified in image fusion step, so some noise suppression process should be done to obtain the enhanced image. The second order PDEs can do high quality denoising, yet the methods tend to cause blocky effects [19]. Fourth order PDEs can avoid the blocky effects. The function is as follows [14]:

(5) $$\begin{array}{c} \frac{\partial P}{\partial t}=-\nabla^{2}\left[\;c\left(\;\left|\;\nabla^{2}P\;\right|\;\right)\nabla^{2}P\;\right]; \end{array}$$

when we get ∂*P*/∂*t*, representing the difference between original image and the denoised imaging containing noise information, we reduced the noise information to get the denoising, where *c*(|∇*P*|) is as follows [14]:

(6) $$\begin{array}{c} c\left(\;\left|\;\nabla P\;\right|\;\right)=\frac{1}{1+{\left(\left|\nabla P\right|/k\right)}^{2}}, \end{array}$$

where *k* was set to be 1. This step can be run more than one time if necessary.

The image processed by the fourth order PDEs may also reserve some noise, so the relaxed median filter can be used to smooth the image further [15]. Assuming that the image processed by the fourth order PDEs can be represented as *P*_pde_, the function is given as follows [15]:

(7) Prelax_m=RMα,ωWi,j=Ppdei,jif  Ppdei,j∈Wi,jαWi,jω,Wi,jmotherwise,

where [*W*_(*i*,*j*)_]_(*m*)_ is the median value of the samples inside the window *W*_(*i*,*j*)_. And the sliding window *W*_(*i*,*j*)_ at the window located at the point is as follows:

(8) $$\begin{array}{c} W_{\left(i,j\right)}=\left\{\;P_{\text{pde}\left(i+r,j+r\right)}:r\in W\;\right\}. \end{array}$$

## 3. Results and Discussion

### 3.1. Testing Data Sets

In this section, three public databases are used to test the proposed method, namely, the DRIVE database [20], the STARE database [21], and the DIARETDB1 database [22]. The DRIVE database, originally collected by Staal et al. [20], contains 40 color retinal images with the size of 565 × 584 pixels. This database is divided into two sets—a training set and a testing set, which is captured using the 45-degree field-of-view digital fundus camera. The STARE database, originally collected by Hoover et al. [21], contains 20 color retinal images with the size of 700 × 605 pixels, which is captured using the 35-degree field-of-view digital fundus camera. The DIARETDB1 database, originally collected by Kauppi et al. [22], contains 89 color retinal images with the size of 1500 × 1152 pixels, which is captured using the 50-degree field-of-view digital fundus camera.

### 3.2. Parameter Determination

There are several important parameters directly affecting the enhancement results. There are two parameters, *σ*_*s*_ and *σ*_*r*_, in (2) of the normalized convolution with a domain transform, and there is one parameter *a* in (4) of the image fusion.

**Figure 1:**
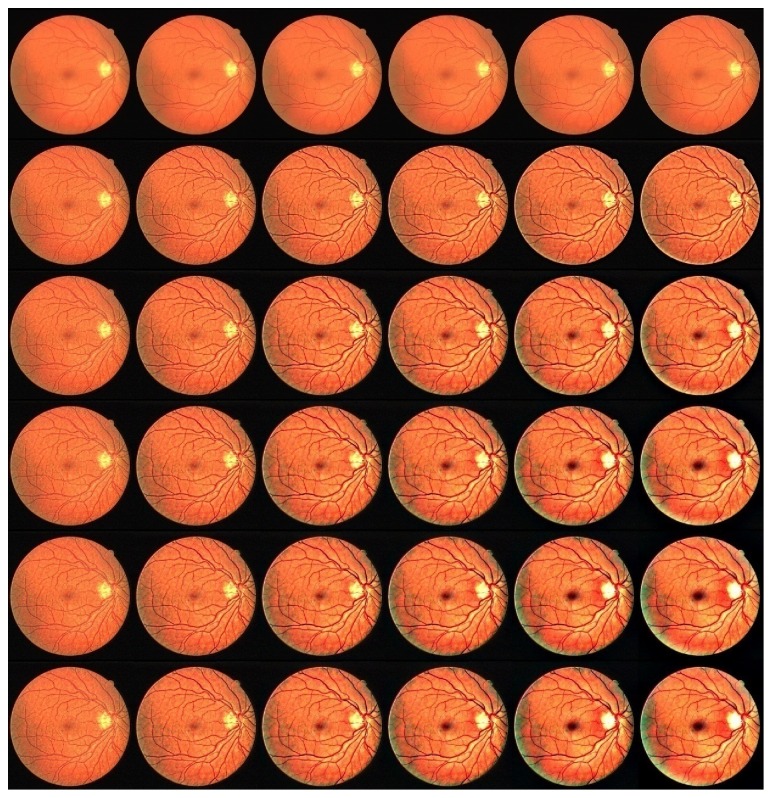
The enhancement results of the retinal image with different *σ*_*s*_ and *σ*_*r*_.

In order to determine the parameters *σ*_*s*_ and *σ*_*r*_ in (2), we changed the values of *σ*_*s*_ and *σ*_*r*_ to test which values are suitable for retinal image enhancement. As the characteristic of retinal image is relatively stable, an original retinal image which is randomly chosen from the DRIVE database is enhanced by the domain transform and the normalized convolution algorithm with different values of *σ*_*s*_ and *σ*_*r*_ (*σ*_*s*_ = 1, 10, 30, 60, 100, 150 and *σ*_*r*_ = 0.1, 0.2, 0.4, 0.6, 1, 1.5). Here, *a* is set as 5 randomly. In Figure 1, the parameter *σ*_*r*_ changes by column, and the parameter *σ*_*s*_ changes by row. From the bottom right corner of Figure 1, we can see that, as the parameters *σ*_*s*_ and *σ*_*r*_ increase, the background of the retinal image is overenhanced gradually and the vessels in the optic disk are missing gradually. As a compromise, we choose *σ*_*s*_ as 60 and *σ*_*r*_ as 0.4 in this paper.

**Figure 2:**
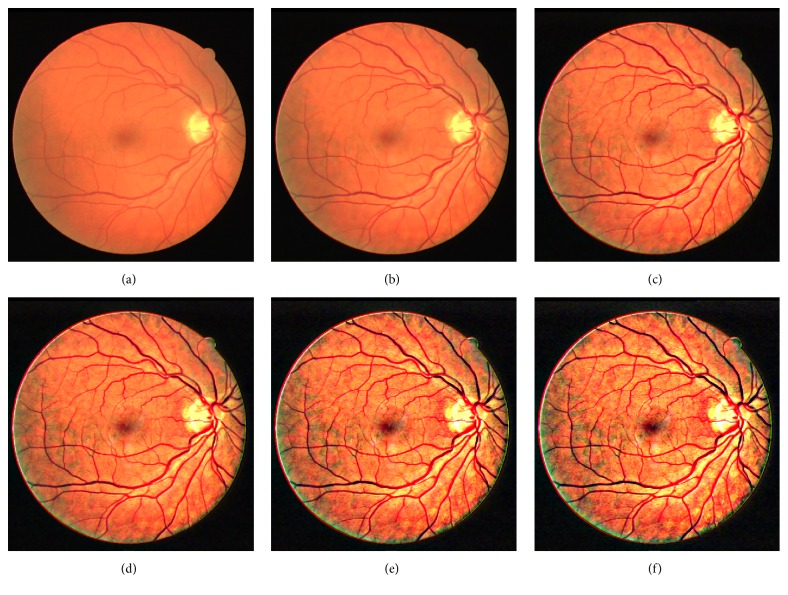
The enhancement results of the retinal image with different *a*. (a) Original image; (b) enhanced image with *a* = 1; (c) enhanced image with *a* = 3; (d) enhanced image with *a* = 5; (e) enhanced image with *a* = 8; (f) enhanced image with *a* = 10.

In order to determine the parameter *a* in (4), we varied the value of *a* to test which value is suitable for retinal image enhancement. The original retinal image which is randomly chosen from the DRIVE database is enhanced by the domain transform and the normalized convolution algorithm with different value of *a* (*a* = 1, 3, 5, 8, 10). Here, *σ*_*s*_ and *σ*_*r*_ are set as 60 and 0.4.  Figure 2(a) is an original retinal image and Figures 2(b)–2(f) are the enhanced images with the different value of *a* (*a* = 1, 3, 5, 8, 10). Figures 2(b)-2(c) showed that some dim regions of the original image are not well enhanced. In Figures 2(e)-2(f), the image is overenhanced and the vessel regions are very clear, but many noises are also produced. The image and the vessel regions are efficiently enhanced in Figure 2(d), and fewer noises are produced. So the enhancement result of Figure 2(d) may be a better result, so we let *a* = 5.

### 3.3. Results of Enhancement

**Figure 3:**
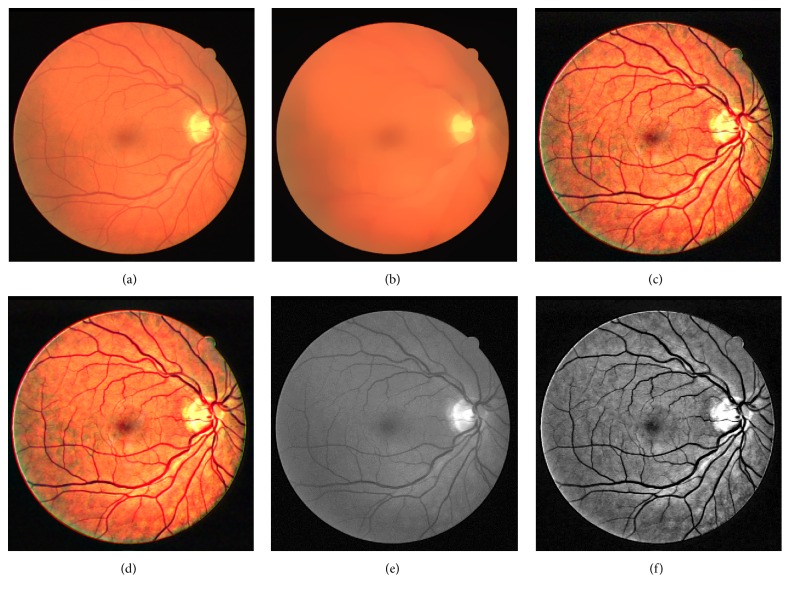
The enhancement process of the chosen retinal image from the DRIVE database. (a) Original image; (b) normalized convolution result; (c) fusion result; (d) denoising result; (e) green channel of original image; (f) green channel of denoising result.

Figure 3(a) is the original retinal image which is randomly chosen from the DRIVE database.  Figure 3(b) is the background image obtained by normalized convolution with a domain transform with the parameter *σ*_*s*_ being 60 and *σ*_*r*_ being 0.4.  Figure 3(c) is the image fusing result with the parameter *a* being 5.  Figure 3(d) is the noise removal result.  Figure 3(e) is the green channel of Figure 3(a), and Figure 3(f) is the green channel of Figure 3(d).

Comparing Figure 3(d) with Figure 3(a) shows that the enhanced image improved image contrast level. The details of the small vessels are enhanced significantly. The dim region such as fovea becomes much dimmer.

**Figure 4:**
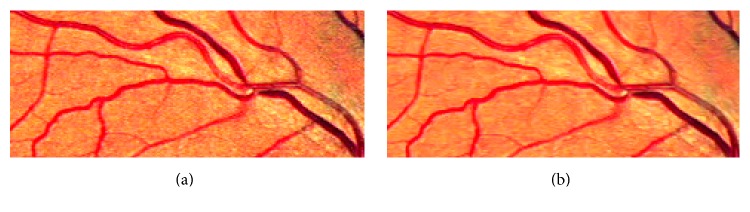
The subregion of Figure 3(c) (before denoising) and Figure 3(d) (after denoising). (a) Subregion of Figure 3(c); (b) subregion of Figure 3(d).

The subregions of the images in Figure 3 are used to show the denoising effects.  Figure 4(a) is the subregion of Figure 3(c), and Figure 4(b) is the subregion of Figure 3(d).  Figure 4 shows that the noise produced by image enhancement steps is depressed effectively.

**Figure 5:**
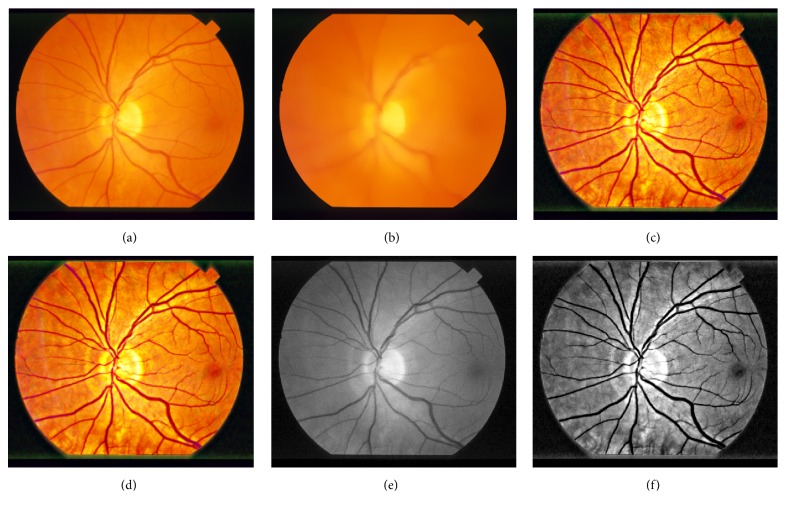
The image enhancement results of an image selected from the STARE database. (a) Original image; (b) normalized convolution result; (c) fusion result; (d) denoising result; (e) green channel of original image; (f) green channel of denoising result.

**Figure 6:**
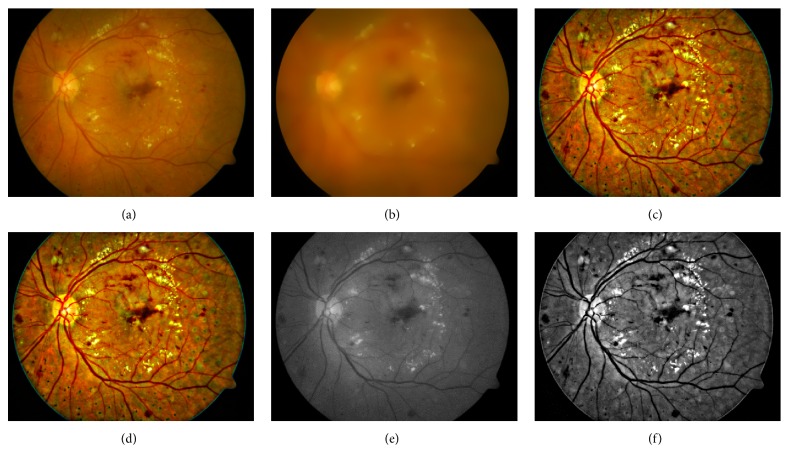
The image enhancement results of an image selected from the DIARETDB1 database. (a) Original image; (b) normalized convolution result; (c) fusion result; (d) denoising result; (e) green channel of original image; (f) green channel of denoising result.

Figures 5 and 6 show the image enhancement results of a randomly selected image from the STARE database and the DIARETDB1 database, respectively. Figures 5(a)–5(f) are the original image from the STARE database, the normalized convolution result, the fusion result, the denoising result, the green channel of original image, and the green channel of denoising result, respectively. Similarly, Figures 6(a)–6(f) are the original image from the DIARETDB1 database, the normalized convolution result, the fusion result, the denoising result, the green channel of original image, and the green channel of denoising result, respectively. These results show that the proposed method has wide applicability.

### 3.4. Evaluating Image Enhancement Effects by Comparing with Other Methods

There are two main ways to evaluate image enhancement effects: one is subjective evaluation and the other is objective evaluation. The subjective evaluation is done by observing the enhancement by experts or persons with image evaluation experience. The advantage of subjective evaluation is that the evaluation results are more in line with the purpose of image enhancement, but the disadvantage is that the subjective evaluation may vary from person to person, and it is a problem how to quantify the evaluation. The objective evaluation methods can quantitatively evaluate the image enhancement results, but it is difficult for the indicator of the objective evaluation methods to reflect human's subject perception. So we compare the image enhancement results with other methods using both the subjective evaluation and the objective evaluation methods to evaluate the image enhancement results.

As vessel is important information in retinal fundus image, two objective evaluating methods are used to quantify the quality of the enhanced image. One method is used to quantify the enhancement of the retinal vessels, and the other is used to quantify the enhancement of the whole image.

The first objective evaluating method tends to quantify the enhancement of the retinal vessels. The measure is called the contrast improvement index [7], which is defined as follows:

(9) $$\begin{array}{c} \text{CII}=\frac{C_{en}}{C}, \end{array}$$

where *C*_en_ and *C* are the contrast values for the retinal vessel in the enhanced image and the original image, respectively. Here, the values of *C*_en_ and *C* are calculated without the pixels of the black background points outside the pupil. Here, *C* is defined as follows:

(10) $$\begin{array}{c} C=\left|\;\frac{f-b}{f+b}\;\right|, \end{array}$$

where *f* and *b* are the average gray values of the retinal vessels and the non-vessel regions, respectively. It is obvious that a larger value of *C* means a larger difference between the retinal vessels and the non-vessel regions. *C*_en_ can be calculated in the same way as *C*, and the difference is just the fact that the image used to produce *C*_en_ is the enhanced image. It should be pointed out that the manually segmented retinal vessels of the original image should be available using this method.

The second objective evaluating method used to quantify the enhancement of the whole image. The measure is called linear index of fuzziness [10], which is defined as follows:

(11) rfen=2MN∑x=1m∑y=1nmin⁡pxy,1−pxy,pxy=sin⁡π2×1−fx,yfmax,

where *f*_max_ is the maximum value of the whole image with size *M* × *N*.

A larger value of CII shows that the retinal vessels are enhanced better. And a smaller value of *r* shows that the whole enhanced image is clearer and has less noise. Therefore, a large value of CII and a small value of *r indicate a good image enhancement method*.

The image enhancement results of the proposed method are color images. Yet, the green channel image is generally used to evaluate image enhancement results. So only the green channel of the enhanced image is used in image evaluation step.

**Figure 7:**
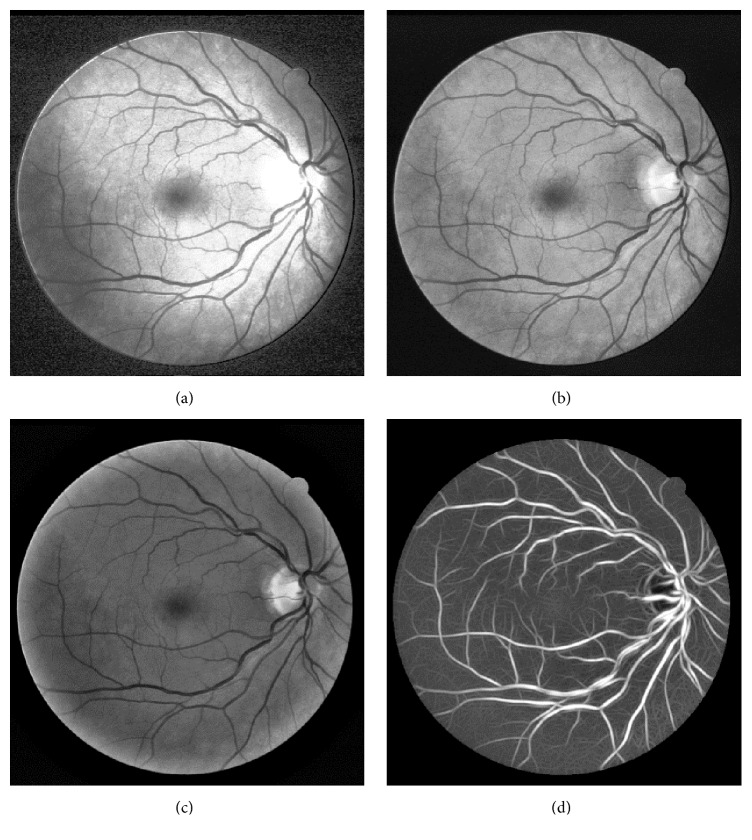
The enhancement results with different methods. (a) The enhancement result using HE; (b) the enhancement result using CLAHE; (c) the enhancement result using method in [7]; (d) the enhancement result using method in [9].

The image enhancement results of Figure 3(e) with other methods, such as HE [3], CLAHE [4], and the methods in [7, 9], are shown in Figure 7. Figures 7(a)–7(d) are the enhancement result using HE, the enhancement result using CLAHE, the enhancement result using method in [7], and the enhancement result using method in [9], respectively.  Table 1 shows the comparison of CII and *r* corresponding to different image enhancement results of Figure 3(e). It is shown that the proposed method has a larger value of CII and a relatively smaller value of *r* comparing with the other methods. It should be pointed out that before calculating CII and *r* of the image shown in Figure 7(d) it is inverted for the purpose of comparing all the methods at the same condition that the vessels appear darker than the background.

**Figure 8:**
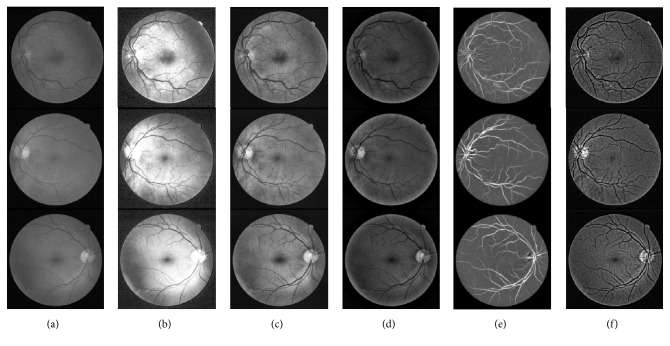
The comparison of image enhancement results of three images in the DRIVE database. (a) The original image; (b) the enhancement results of HE; (c) the enhancement results of CLAHE; (d) the enhancement results using the method in [7]; (e) the enhancement results using the method in [9]; (f) the enhancement results of the proposed method.

Three retinal images randomly chosen from the DRIVE database are also used to compare image enhancement results shown in Figure 8. The first column (Figure 8(a)) is the green channel of each original image. The middle four columns (Figures 8(b)–8(e)) are the enhancement results of the HE, the CLAHE, and the methods in [7, 9], respectively. The last column is the results of the proposed method. From the comparison of image enhancement results, we can see that the HE method produces uneven background image which changed the illumination distribution of the original image, and the low-contrast region is not enhanced well. So many details would be missed by using the HE method as shown in Figure 8(b). The CLAHE method could enhance the details of the retail image, but the whole contrast is not improved significantly as shown in Figure 8(c). The results of using the method in [7] are too dim as shown in Figure 8(d). The image enhancement results using the method in [9] lose the details of the background as shown in Figure 8(e). The values of CII and *r* of each image in Figure 8 are shown in Table 2. It shows that the retinal vessels are enhanced better and the whole image is clearer using the proposed method as shown in Figure 8(f).

**Figure 9:**
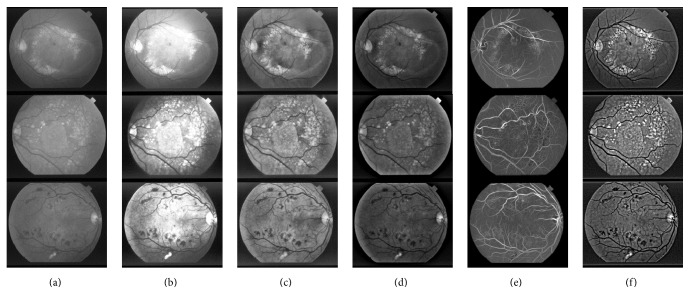
The comparison of image enhancement results of three images in the STARE database. (a) The original image; (b) the enhancement results of HE; (c) the enhancement results of CLAHE; (d) the enhancement results using method in [7]; (e) the enhancement results using method in [9]; (f) the enhancement results of the proposed method.

The image enhancement results for pathological retinal fundus images chosen from the STARE database are shown in Figure 9. The first column (Figure 9(a)) is the green channel of each original image. The middle four columns (Figures 9(b)–9(e)) are the enhancement results of the HE, the CLAHE, and the methods in [7, 9], respectively. The last column is the results of the proposed method as shown in Figure 9(f). It shows that the proposed method not only enhances the retinal vessels and makes the whole image clearer, but also enhances the pathological regions well. The values of CII and *r* of each image in Figure 9 are shown in Table 3. It shows that the proposed method could perform better than the other methods on the pathological images.

**Figure 10:**
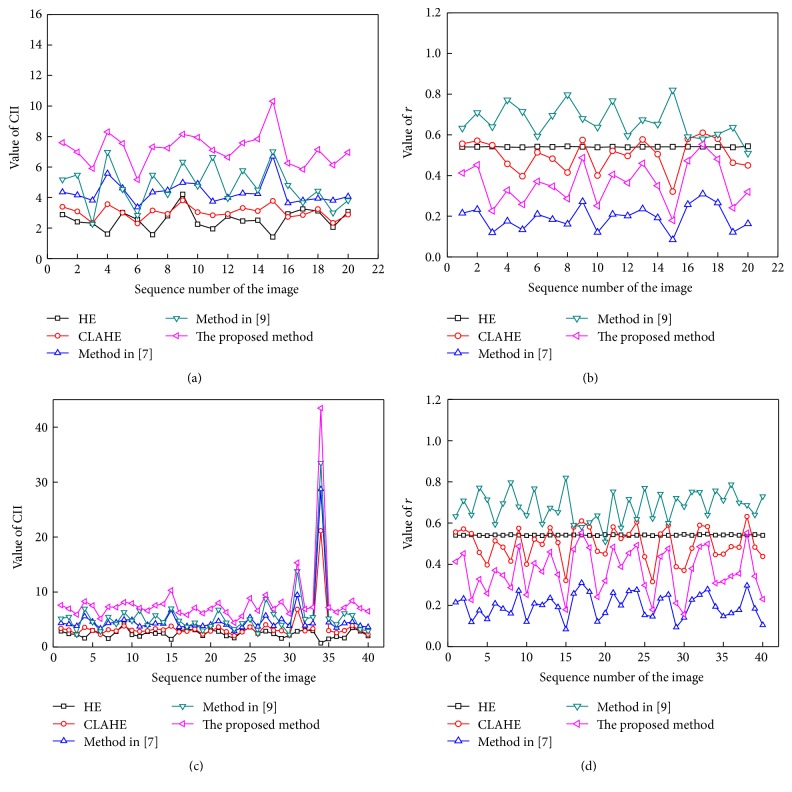
Comparison of the line charts of CII and* r* on the DRIVE database. (a) The line charts of CII of the testing set; (b) the line charts of *r* of the testing set; (c) the line charts of CII of the testing and training sets; (d) the line charts of* r* of the testing and training sets.

**Figure 11:**
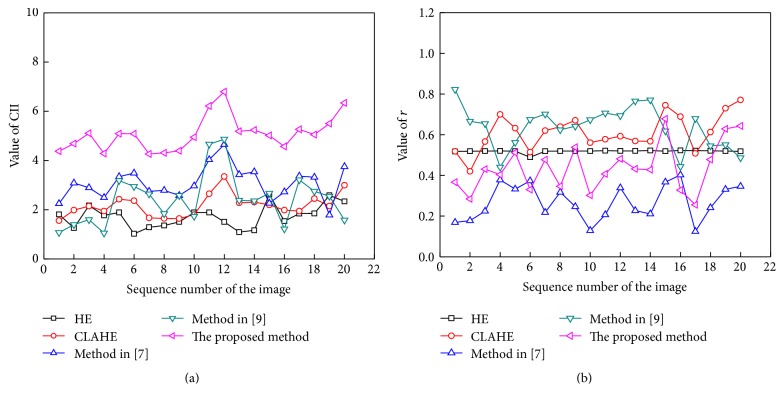
Comparison of the line charts of CII and *r* on the STARE database. (a) The line charts of CII; (b) the line charts of *r*.

The comparison of line charts of CII and *r* values for the images of DRIVE database is shown in Figure 10. Figures 10(a)-10(b) are the results using the 20 images in the testing set of DRIVE database. And Figures 10(c)-10(d) are the results using all the 40 images of DRIVE database including testing set (20 images) and training set (20 images). In Figures 10(a)–10(d), the *x*-axis denotes the sequence number of the image, and the *y*-axis denotes the value of CII and *r* of the different methods. By the same token, Figures 11(a) and 11(b) show the line charts of the values of CII and *r* of the different methods used to enhance retinal fundus images of the STARE database, respectively.

It is shown from Figures 10 and 11 that the proposed method achieved the largest CII, which indicates that the proposed method is the best in enhancing the retinal vessels. And the value of *r* of the proposed method is nearly second small comparing with the other methods, which indicates that the whole image is comparatively clearer after enhancement using the proposed method. The enhancement performance of the proposed method on both normal and pathological retinal images is comparatively better.

The proposed method shows some merits comparing with other methods. (1) The edges of the vessels change softly and do not cause artificial edges as there are not abrupt changes in the image containing basic information. (2) As the enhanced image is obtained by fusing the image with basic information of the background and the original image, there is no main detail losing. (3) A proper noise reduction filter is used to depress the noise caused by the fusing step.

The running times of different methods were calculated on a computer with Windows 7 OS, a Dual Core 3.40 GHz CPU, 4 GB of RAM, under Matlab R2012 software. The average running time comparisons of different methods on the DRIVE database and STARE database are shown in Tables 4 and 5, respectively. It is shown from the two tables that the fastest method is the HE method and the most time consuming method is the proposed method on both the DRIVE database and STARE database. The proposed method needs to do some time consuming processes such as domain transform, convolution, the fourth order PDEs, and the relaxed median filter to achieve image enhancement, which makes the proposed method consume much more average running time than all the other methods. As running time is also a very important factor in clinical application, this is an obvious defect of the proposed method. In the future, if we can find another fast image denoising method suitable for removing the noise caused by image fusion, the running time may be reduced to some extent.

## 4. Conclusions

In this paper, a retinal image enhancement method fusing original image and the image containing the basic information of the background is presented. The proposed method is tested on the retinal images of the DRIVE database, the STARE database, and the DIARETDB1 database, respectively. Two objective evaluation indexes (CII and* r*) which could measure both the contrast and clarity of the enhanced image are used. The CII value of the proposed method is distinctly the largest and the *r* value is the second smallest in all the compared methods on the DRIVE and the STARE databases. It is shown that the proposed method achieves a much better performance on enhancing retinal fundus image comparing with the other methods. Moreover, different from other image enhancement methods mentioned above, the proposed method can handle color images which may be more beneficial to diagnosis by the ophthalmologists.

## Figures and Tables

**Table 1:** The comparison of CII and *r* with different methods on the image shown in Figure 3(a).

Evaluation measures	HE	CLAHE	Method in [7]	Method in [9]	The proposed method
CII	2.3981	3.0866	4.1526	5.4816	6.9785
*r*	0.5406	0.7402	0.4453	0.7094	0.4523

**Table 2:** The comparison of CII and *r* with different methods on the images shown in Figure 8.

Image	Evaluation measures	HE	CLAHE	Method in [7]	Method in [9]	The proposed method
1st in column (a)	CII	2.3105	2.3252	3.8081	2.2630	5.9076
	*r*	0.5391	0.6264	0.2012	0.6401	0.2267

2nd in column (a)	CII	3.0113	3.0059	4.6100	4.5377	7.5447
	*r*	0.5418	0.5454	0.2979	0.7148	0.2582

3rd in column (a)	CII	2.2450	3.0392	4.8968	4.7682	7.9369
	*r*	0.5412	0.4865	0.2004	0.6378	0.2514

**Table 3:** The comparison of CII and *r* with different methods on images shown in Figure 9.

Image	Evaluation measures	HE	CLAHE	Method in [7]	Method in [9]	The proposed method
1st in column (a)	CII	1.2610	1.9859	3.0856	1.3910	4.6879
	*r*	0.5189	0.4203	0.1781	0.6664	0.2845

2nd in column (a)	CII	2.1659	2.1467	2.8935	1.5957	5.1086
	*r*	0.5196	0.5660	0.2247	0.6549	0.4306

3rd in column (a)	CII	1.8862	1.7994	2.9650	1.7290	4.9442
	*r*	0.5196	0.5607	0.1294	0.6735	0.3019

**Table 4:** The average running time comparison of different methods on the DRIVE database (including 40 images).

Evaluation methods	HE	CLAHE	Method in [7]	Method in [9]	The proposed method
Time/s	0.012	0.045	0.291	2.672	18.152

**Table 5:** The average running time comparison of different methods on the STARE database (including 20 images).

Evaluation methods	HE	CLAHE	Method in [7]	Method in [9]	The proposed method
Time/s	0.006	0.044	0.359	3.406	23.788
